# Global research trends and focus on the link between colorectal cancer and gut flora: a bibliometric analysis from 2001 to 2021

**DOI:** 10.3389/fmicb.2023.1182006

**Published:** 2023-05-05

**Authors:** Yonglong Chang, Qinling Ou, Xuhui Zhou, Jinhui Liu, Sifang Zhang

**Affiliations:** ^1^Department of Integrated Traditional Chinese and Western Medicine, The Second Xiangya Hospital, Central South University, Changsha, China; ^2^National Clinical Research Center for Metabolic Diseases, Changsha, China; ^3^Department of Addiction Medicine, Hunan Institute of Mental Health, Brain Hospital of Hunan Province (The Second People’s Hospital of Hunan Province), Changsha, China; ^4^College of Integrated Traditional Chinese and Western Medicine, Hunan University of Traditional Chinese Medicine, Changsha, China

**Keywords:** colorectal cancer, gut microbiota, bibliometric analysis, research trends, CiteSpace, VOSviewer

## Abstract

**Background:**

Colorectal cancer (CRC) is a highly prevalent cancer, and the global healthcare system bears a significant burden due to its incidence. Modulating the gut microbiota is a promising approach to enhance the efficacy of CRC treatment and reduce its adverse effects. The causal relationship between specific microorganisms’ presence and CRC development has been widely validated. However, few studies have investigated this relationship using bibliometric methods. Therefore, this study analyzed the research hotspots and trends in human gut microbiology and CRC over the last two decades from a bibliometric perspective. The study aims to provide novel insights into basic and clinical research in this field.

**Methods:**

The articles and reviews on gut microbiota in CRC were obtained from the Web of Science Core Collection (WOSCC) on November 2, 2022. CiteSpace and VOSviewer were used to conduct the bibliometric and knowledge-map analysis.

**Results:**

A total of 2,707 publications were obtained, with a rapid increase in the number of publications since 2015. The United States and China are the main contributors in this field and have established a network of partnerships in several countries. 414 academic journals have published articles on this topic. The author with the highest number of publications is Jun Yu from the Chinese University of Hong Kong. In addition to “intestinal flora” and “colorectal cancer,” high frequency terms in the keyword co-occurrence network analysis included inflammatory bowel disease, *Fusobacterium nucleatum*, inflammation, long-chain fatty acids, ulcerative colitis, bile acids, and resistant starch. Analysis of keyword trends using burst testing revealed that biomarkers, abnormal crypt foci, bifidobacteria, β-glucuronidase, short-chain fatty acids, bile acids, and DNA methylation are at the forefront of research in this area.

**Conclusion:**

The findings of this study provide a bibliometric analysis and visualization of the key research areas in gut microbiota and CRC over the past 20 years. The results suggest that the role of gut microbiota in CRC and its underlying mechanisms should be closely monitored, particularly in the areas of biomarkers, metabolic pathways, and DNA methylation, which may emerge as hot topics in this field.

## Introduction

Colorectal cancer (CRC) is the second leading cause of cancer-related morbidity and mortality worldwide (Wong and Yu, 2019). The etiology of CRC is complex and involves multiple genes and signaling pathways. It is estimated that the number of new CRC cases will increase to 2.5 million globally by 2035, surpassing more common cancers such as gastric and liver cancers. In the past few years, several studies have demonstrated the association of environmental and genetic factors with CRC (Yu et al., 2015), and gut microbes are considered to play an important role in the development of CRC (Cai et al., 2022).

Gut microbes, a large community of microorganisms residing in the intestine, were first studied by the American scholar Luckey in 1972. It is estimated that there are about 10 trillion bacteria in the human bowel, and these bacteria constitute a complex dynamic system that plays an important role in regulating human health functions such as enhancing autoimmunity and fighting off infections (Zmora et al., 2018). Based on the role of intestinal flora in human health, they can be divided into three categorie (Park et al., 2022). The first category is beneficial bacteria, also known as probiotics, mainly including *bifidobacteria*, *lactobacilli*, etc. They are indispensable elements of human health and can synthesize several vitamins, participate in the digestion of food, promote intestinal peristalsis, inhibit the growth of pathogenic flora, decompose harmful and toxic substances, etc. The second category is harmful bacteria, such as *Fusobacterium nucleatum*, Enterotoxigenic *Bacteroides fragilis* (ETBF), *Helicobacter pylori*, etc. Once these bacteria grow and multiply in large numbers, they can lead to several diseases, producing harmful substances such as carcinogens or affecting the immune system functioning. The third category is neutral bacteria, which is a category of bacteria performing dual roles, such as *E. coli, enterococci,* etc. They play important roles in human health under normal circumstances, but once they proliferate out of control or are transferred from the intestine to other parts of the body, they may lead to several health problems. Human health is closely related to the composition of probiotic flora in the intestinal tract. During the long-term evolutionary process of host adaptation and natural selection, the intestinal flora is always in a dynamic balance between the different species of flora and the host and between the host and its environment, which constitutes an interdependent and mutually constrained ecosystem. Furthermore, the material metabolism and energy conversion processes in the intestine involving digestion and absorption of food can provide nutrition for the host and the organism. However, the disruption of the aforementioned dynamic balance can cause damage to human health. According to a previous study, dysbiosis of intestinal flora can lead to micro-ecological changes, which in turn, can lead to inflammatory bowel disease (IBD), CRC, and several other disease (Cheng et al., 2020).

Over the past few decades, a large number of scientific studies have demonstrated the relationship between gut flora and CRC. These studies were mainly based on the pathways by which gut flora dysbiosis contributes to the pathogenesis of CRC, the clinical significance of gut flora in CRC, and the investigation of the mechanisms underlying the action of gut flora in CRC based on the bioinformatics techniques such as metagenomics. Although the exact mechanism underlying the role of gut flora in the development of CRC is still unclear, we have summarized it based on the existing research literature. The mechanism primarily includes three aspects: firstly, it causes DNA damage in intestinal epithelial cells by producing toxins (Dejea et al., 2018; Li et al., 2019; Chen et al., 2022) secondly, it causes metabolic disorders in the body by forming secondary metabolites (Taira et al., 2015; Paik et al., 2022; Ryu et al., 2022); and finally, it changes the tumor microenvironment and promotes tumor immunosuppression (Kostic et al., 2013; Long et al., 2019). So far, however, very few studies have summarized the research in this field over the past 20 years to help us better analyze the current state and future research trends of gut microbiota and its relationship with CRC. Bibliometrics is an interdisciplinary field that uses mathematical and statistical methods to analyze information from published sources (Ma et al., 2021). It can visualize and present the results so that the researchers can quickly and clearly understand the latest trends in the relevant research fields and provide suggestions for further research. Currently, CiteSpace (Chen, 2006) and VOSviewer (van Eck and Waltman, 2009; Liang et al., 2017) are used primarily for the scientometric analysis of the literature, and several researchers have used this strategy to assess their respective research areas.

This study aims to analyze the hot spots and trends in gut flora and its role in CRC over the past two decades, map the scientific knowledge in this research field, describe the current status of the field (Ma et al., 2021), and provide a basis for future scientific research on the relationship between gut flora and CRC.

## Methods

### Data source and search strategy

Bibliographic data were obtained from the Science Citation Index (SCI) Expanded Database of the Web of Science (WoS). To avoid bias caused by the daily database updates, all the publications from 2000 to 2021 were retrieved and downloaded from the WoS Core Collection (WoSCC) database on November 2, 2022. The search strategy applied was Theme = (“Rectal Neoplasm” OR “Rectal Tumor” OR “Rectal Cancer” OR “Rectum Neoplasm” OR “Rectum Cancer” OR “Cancer of the Rectum” OR “Cancer of Rectum” OR “Colorectal Neoplasm” OR “Colorectal Tumor” OR “Colorectal Cancer” OR “Colorectal Carcinoma” OR “Colonic Neoplasm” OR “Colon Neoplasm” OR “Cancer of Colon” OR “Colon Cancer” OR “Cancer of the Colon” OR “Colonic Cancer” OR “CRC”) AND Theme = (“Intestinal flora” OR “Gastrointestinal Microbiome” OR “Gastrointestinal Microbiomes” OR “Gut Microbiome” OR “Gut Microbiomes” OR “Gut Microflora” OR “Gut Microbiota” OR “Gut Microbiotas” OR “Gastrointestinal Flora” OR “Gut Flora” OR “Gastrointestinal Microbiota” OR “Gastrointestinal Microbiotas” OR “Gastrointestinal Microbial Community” OR “Gastrointestinal Microbial Communities” OR “Gastrointestinal Microflora” OR “Gastric Microbiome” OR “Gastric Microbiomes” OR “Intestinal Microbiome” OR “Intestinal Microbiomes” OR “Intestinal Microbiota” OR “Intestinal Microbiotas” OR “Intestinal Microflora” OR “Enteric Bacteria”). Finally, only the research papers and review articles in English were considered for this study.

**Figure 1:**
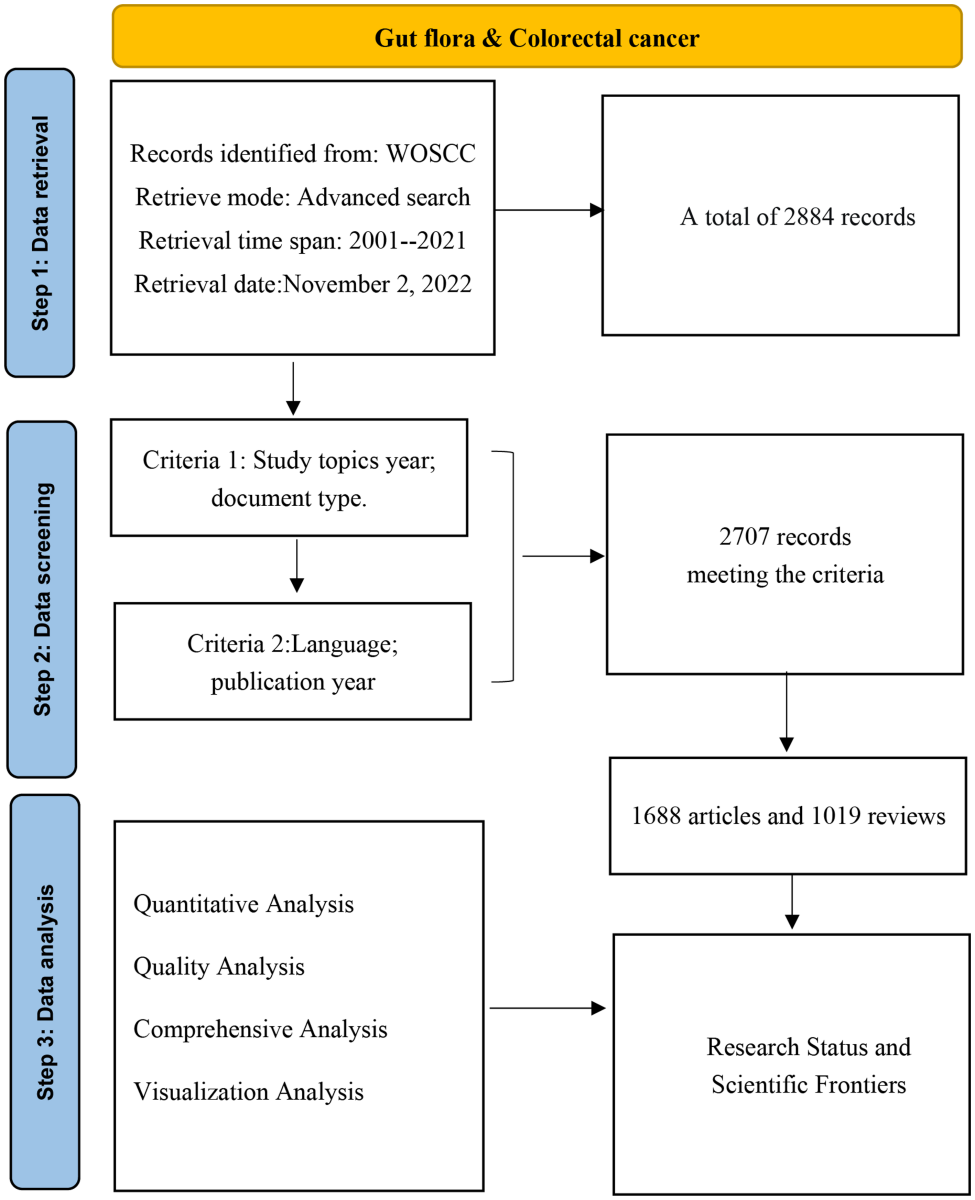
A flowchart of the publication screening process.

Figure 1 is a flow chart of the data screening process, showing the inclusion and exclusion criteria for publications and the methodology and content of the analysis.

### Data analysis

#### Quantitative analysis

The collected literature was analyzed based on four criteria: the number of publications authored by the top 10 authors, the number of publications in the top 10 journals, the number of publications from the top 10 institutions, and the number of publications from the top 10 countries/regions.

#### Quality analysis

The quality analysis was based on the following aspects: authors and co-cited authors, journals and co-cited academic journals, analysis of co-cited references, and analysis of co-cited keywords.

#### Comprehensive analysis

Our analysis includes visualizing and analyzing the collaborative network relationships between different countries, the H-index scores of the top 10 journals and authors, and the current state of research in the field over the past 20 years. Additionally, we also highlight future trends in the field.

#### Visualization analysis

To carry out visual analysis, all the valid data extracted from the WoSCC database were imported into CiteSpace [version 6.1] and VOSviewer [version 1.6.18]. CiteSpace was applied to analyze the strongest citation bursts of references and keywords; investigate the research status, hotspots, and trends; prepare distribution maps over time; and determine the development trends of the field. A visual analysis of the collaborative networks between countries, institutions, journals, and authors and the co-citation of keyword clusters was conducted using VOSviewer. Meanwhile, R (version 4.1) was used for the visual data analysis, including the relationships among authors, institutions, and country collaborations from the bibliometric analysis website.^1^ By examining keyword frequency, degree of centrality, and prominence, we can completely understand the research status, hotspots, and development trends in this field. Based on the co-occurrence of keywords, a keyword co-occurrence network was constructed, with each node representing a keyword. When two keywords appear in an article at the same time, they form a co-occurrence relationship, which is represented as an edge in the network. If a particular subject significantly contributes to this field, it is represented by a node with a high mean value. The degree of occurrence indicated that the crosstalk and the number of co-references of a node increased over time. If the degree of occurrence is greater, it is more evident that the node is a research hotspot during a given period.

## Results

### Annual trends in publications

**Figure 2:**
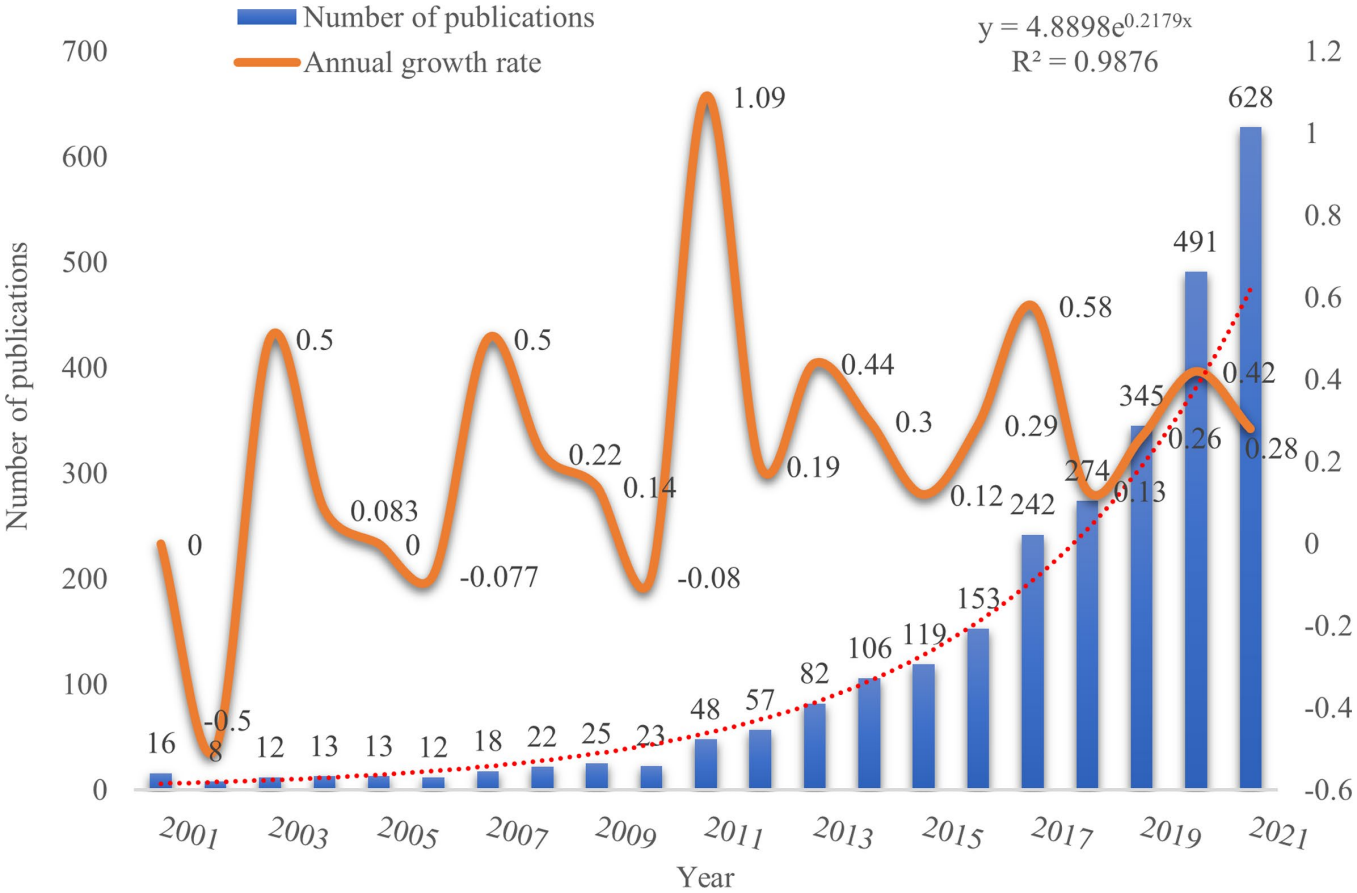
Temporal trend distribution of articles on intestinal flora in CRC field.

The trend and the number of publications in a field can reflect the stages of development in the field and predict the research growth. According to the search criteria and time range set, a total of 2,707 publications related to CRC and gut flora were retrieved from the WoSCC database, among which 1,688 (62.36%) were articles and 1,019 (37.64%) were review papers. Figure 2 shows the trend in the number of annual publications on gut flora in CRC from 2001–2021. We divided this trend into two periods: the slow growth period (2001–2015) and the fast growth period (2015–2021). The growth in the number of publications before 2015 was relatively slow, whereas there was a rapid growth in the number of publications after 2015. When compared to the number of publications before 2015, the number of publications after 2015 exceeded 100 per year and showed an increasing trend in the annual number of publications. The year 2021, with 628 publications, had the highest number of publications on CRC and intestinal flora in the past 10 years. Figure 2 also shows a polynomial curve fitting the total annual growth trend of publications. The results indicated an increasing trend in the annual number of publications, which was highly correlated with the year of publication (*R*^2^ = 0.9876) (Shen et al., 2022). Overall, these findings suggested that research on the relationship between gut flora and CRC had an annually increasing trend, and many researchers were investigating the role of gut flora in CRC.

Several countries/regions have conducted research on the relationship between gut flora and CRC. The top 5 countries in terms of the number of publications were the USA (829 papers), the People’s Republic of China (711 papers), Italy (192 papers), Germany (138 papers), and France (128 papers). Table 1 shows that the top 5 countries in terms of centrality were Belgium (0.67), France (0.43), Singapore (0.25), Brazil (0.24), and Northern Ireland (0.24), which differed from the ranking based on the number of publications. Among institutions, Shanghai Jiao Tong University (54 papers) was the most productive, followed by Harvard Med School (49 papers), Chinese University of Hong Kong (44 papers), Zhejiang University (39 papers), and University of Michigan (38 papers), as shown in Table 2. Table 3 indicates that the top 5 authors in terms of publication output were Yu, Jun (33 papers) from the Department of Medicine and Therapeutics, State Key Laboratory of Digestive Diseases, LKS Institute of Health Sciences, Shenzhen Research Institute, The Chinese University of Hong Kong, followed by Chan, Andrew T (22 papers), Ogino, Shuji (18 papers), Jobin, Christian (16 papers), and Garrett, Wendy S (16 papers).

**Table 1:** Top 10 countries with high centrality.

Rank	Country	Centrality	Rank	Country	Centrality
1	Belgium	0.67	6	Serbia	0.2
2	France	0.43	7	Panama	0.2
3	Singapore	0.25	8	Uruguay	0.2
4	Brazil	0.24	9	Germany	0.19
5	Northern Ireland	0.24	10	Malaysia	0.19

**Table 2:** Top 10 active countries/regions, institutions, and authors.

Rank	Country	Records	Rank	Institution	Records
1	USA	829	1	Shanghai Jiao Tong University	54
2	China	711	2	Harvard Medical School	49
3	Italy	192	3	The Chinese University of Hong Kong	44
4	Germany	138	4	Zhejiang University	39
5	France	128	5	University of Michigan	38
6	England	121	6	Harvard T.H. Chan School of Public Health	35
7	Japan	114	7	Sun Yat-Sen University	35
8	Spain	101	8	Massachusetts Gen Hospital	33
9	Canada	91	9	Dana-Farber Cancer Institute	32
10	India	88	10	University of North Carolina	32

**Table 3:** Top 10 authors in terms of the number of publications and top 10 authors in terms of total citations.

Rank	Author	Records	Co-cited Author	Records
1	Yu Jun	33	Kostic AD	652
2	Chan Andrew T	22	Arthur JC	451
3	Ogino Shuji	18	Louis P	401
4	Jobin Christian	16	Rubinstein MR	394
5	Garrett Wendy S	16	Castellarin M	384
6	Fang Jing yuan	16	Turnbaugh PJ	366
7	Song Ming yang	15	Wu SG	339
8	Cao Hai long	13	Sears CL	329
9	Huttenhower Curtis	11	Qin JJ	325
10	Wang Bang mao	11	Okeefe SJD	302

### Authors and co-cited authors

**Figure 3:**
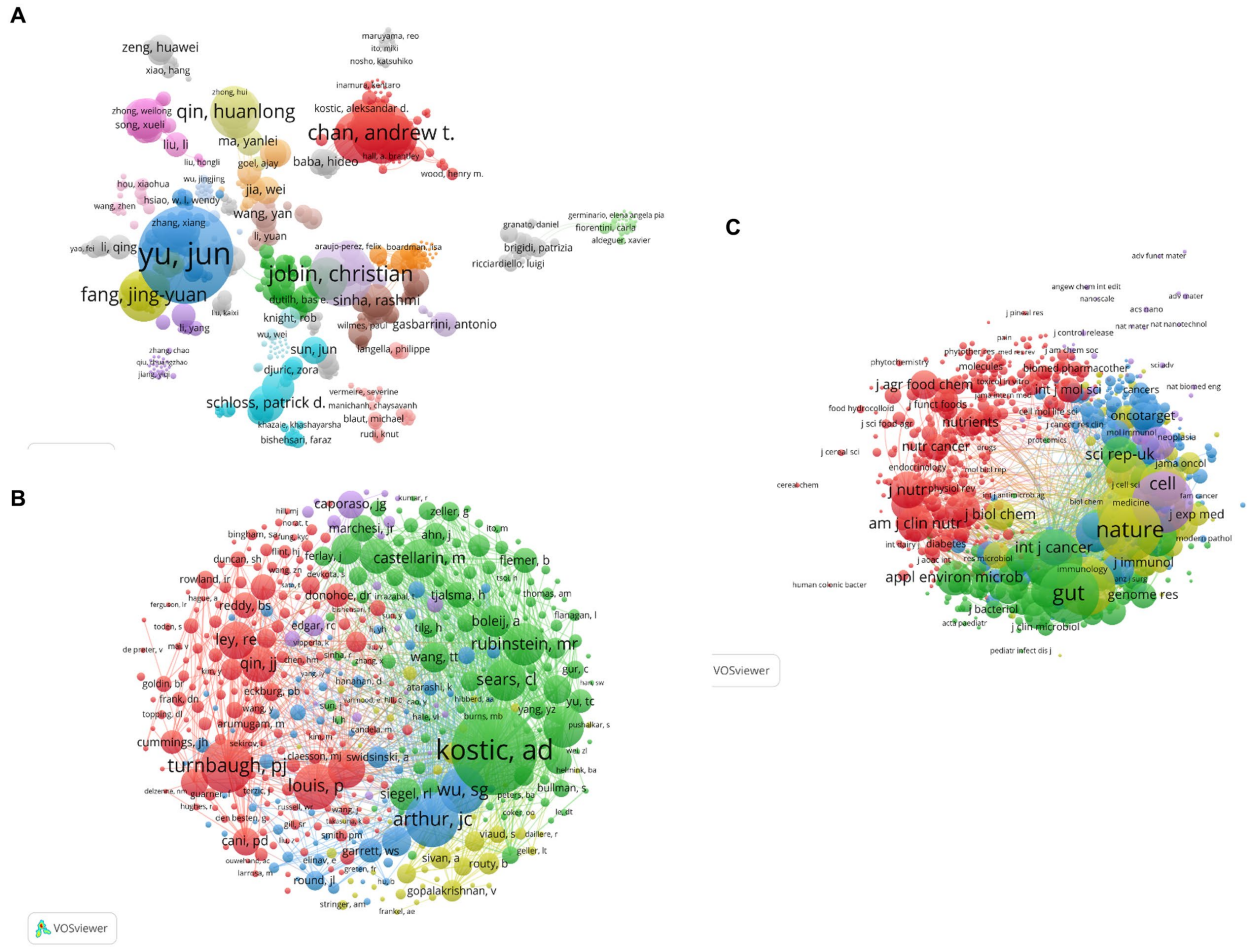
**(A)** VOSviewer visualization analysis of author collaborations, **(B)** VOSviewer-based visualization analysis of author co-citations, and **(C)** VOSviewer visualization analysis of journal publications.

Through the analysis of 2,707 publications, it was found that a total of 13,580 researchers contributed to the study of gut flora in CRC. Figure 3A shows the collaboration network of authors, visualized using VOSviewer. The size of each node represents the number of publications, and the connecting lines indicate collaborations. Larger nodes represent a greater number of publications, and thicker lines indicate closer collaboration. Yu, Jun from the Institute of Digestive Diseases, State Key Laboratory of Digestive Diseases, Department of Medicine and Therapeutics, Shenzhen Research Institute, The Chinese University of Hong Kong had the highest number of publications (33 papers) among all authors. Furthermore, the 13,580 researchers were divided into 26 clusters, with the Yu, Jun cluster and Chan, Andrew T cluster being the two most influential clusters. Interestingly, there was no collaboration between these two largest clusters, indicating the relative independence of research in this field.

CiteSpace was used for the analysis of the author citation information to investigate the academic relationship between authors and visualize them in a network. The network reflects highly cited authors and authors widely recognized by the research community in their field of study. A co-citation occurs when two studies are cited in the same third publication. In this study, 2,707 peer-reviewed publications were considered as the object of analysis, with 392 nodes and 890 links. The size of the node indicates the number of citations received by the scholar, and more links between two nodes indicate more citations of both authors in the same publication. The nodes that are cited more than 200 times are marked by the corresponding first author. As shown in Figure 3B, Kostic, A.D. from Harvard Medical School, USA, was the author who had been co-cited more than once. The top 10 most co-cited authors are provided in Table 3.

### Journals and co-cited academic journals

**Figure 4:**
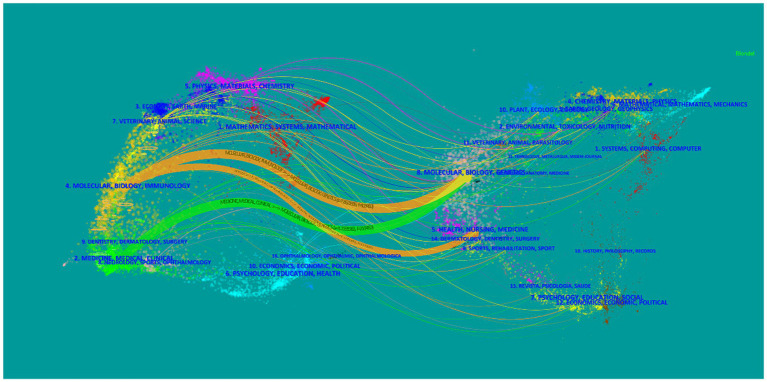
A dual map overlay of journals related to the role of gut flora in CRC.

In total, 414 academic journals published articles related to CRC and gut microbiota, with PLOS ONE (1915 papers, IF 2022 = 3.752) ranking first, closely followed by Gut (1883 papers, IF 2022 = 31.793). Table 4 shows the top ten journals in terms of the number of publications, out of which 70% were from the USA and 20% were from the UK. The journal with the highest impact factor in 2022 was Nature from the UK. As shown in Figure 3C, VOSviewer was applied to analyze the co-cited journals and generate a visual network for determining the journals having a greater impact on the development of the field, where the size of the nodes is proportional to the number of citations, and a positive citation relationship is observed between different academic journals. The dual map overlay is an analytical method that shows domain-level citation concentration with their reference paths. As shown in Figure 4, citing journals are on the left side of the map, while cited journals are on the right side. The labels represent the disciplines covered by the journals, and the colored lines show the citation paths. The dataset in this study contains three major primary citation paths. Two of the orange paths indicate that molecular/biology/immunology journals are frequently cited for research in molecular/biology/genetics journals and health/nursing/medicine journals. The green path indicates that research in medical/clinical journals is frequently cited by research in molecular/biology/genetics journals.

**Table 4:** Top 10 journals in terms of the number of published papers.

Rank	Journal	Records	Centrality	Country	IF (2022)
1	Plos one	1,915	0.06	USA	3.752
2	Gut	1,883	0.09	UK	31.793
3	Nature	1,734	0.11	UK	69.504
4	Gastroenterology	1,714	0.14	USA	33.883
5	Science	1,677	0.02	USA	63.714
6	Proceedings of the national Academy of sciences	1,585	0.02	USA	12.779
7	Cell	1,333	0.04	USA	66.85
8	Cancer research	1,273	0.01	USA	13.312
9	World journal of gastroenterology	1,235	0.01	China	5.374
10	Cell host and microbe	1,170	0.04	USA	31.316

### Analysis of co-cited references

The co-citation analysis was performed on literature spanning from 2001 to 2021, with data collected in time slices. The top 50 frequently cited publications were extracted from each time slice for co-citation network analysis, resulting in a final co-citation network of 781 publications with 1,528 link lines. A modularity value of 0.766 was obtained, indicating a clear delineation of clusters, and 110 clusters were identified. Table 5 presents the top 10 cited papers, with Yu et al.’s “Nucleated *Fusobacterium nucleatum* promotes chemotherapy resistance in CRC by regulating Autophagy” being the most cited (*n* = 237). The paper, which was based on a study evaluating the effect of gut flora on chemoresistance in CRC patients, presented three key conclusion (Yu et al., 2017). Firstly, specific gut microbes are associated with recurrence after CRC chemotherapy. Secondly, *F. nucleatum* controls CRC chemoresistance by regulating Toll-like receptors, microRNAs, and autophagy networks. Finally, regular monitoring and targeting of *F. nucleatum* can contribute to the prognosis and management of CRC patients. Of the 10 high-cited publications, 90% had impact factors of 30 or more, indicating strict quality control in high-quality academic journals. Notably, four of the publications had corresponding authors from the USA and three from China, highlighting the high quality of publications from these countries.

**Table 5:** Top 10 cited publications.

Rank	Co-cited reference	Total Citations	Centrality	Year	Journal	IF (2022)	Corresponding author’s country
1	*Fusobacterium nucleatum* Promotes Chemoresistance to Colorectal Cancer by Modulating Autophagy	237	0.04	2017	Cell	66.85	China
2	Global cancer statistics 2018: Globocan estimates of incidence and mortality worldwide for 36 cancers in 185 countries	194	0	2018	CA: A Cancer Journal for clinicians	286.13	USA
3	Gut microbiome influences efficacy of PD-1-based immunotherapy against epithelial tumors	188	0.02	2018	Science	63.714	France
4	Metagenomic analysis of faecal microbiome as a tool towards targeted non-invasive biomarkers for colorectal cancer	180	0.03	2017	Gut	31.793	China
5	The gut microbiota, bacterial metabolites and colorectal cancer	174	0.04	2014	Nature reviews mcrobiology	78.297	UK
6	*Fusobacterium nucleatum* potentiates intestinal tumorigenesis and modulates the tumor-immune microenvironment	173	0.04	2013	Cell host and microbe	31.316	USA
7	Analysis of Fusobacterium persistence and antibiotic response in colorectal cancer	172	0	2017	Science	63.714	USA
8	Gut microbiome development along the colorectal adenoma-carcinoma sequence	167	0.03	2015	Nature communications	17.694	China
9	*Fusobacterium nucleatum* in colorectal carcinoma tissue and patient prognosis	163	0.04	2016	Gut	31.793	USA
10	Gut microbiota imbalance and colorectal cancer	160	0.03	2016	World journal of gastroenterology	5.374	France

**Figure 5:**
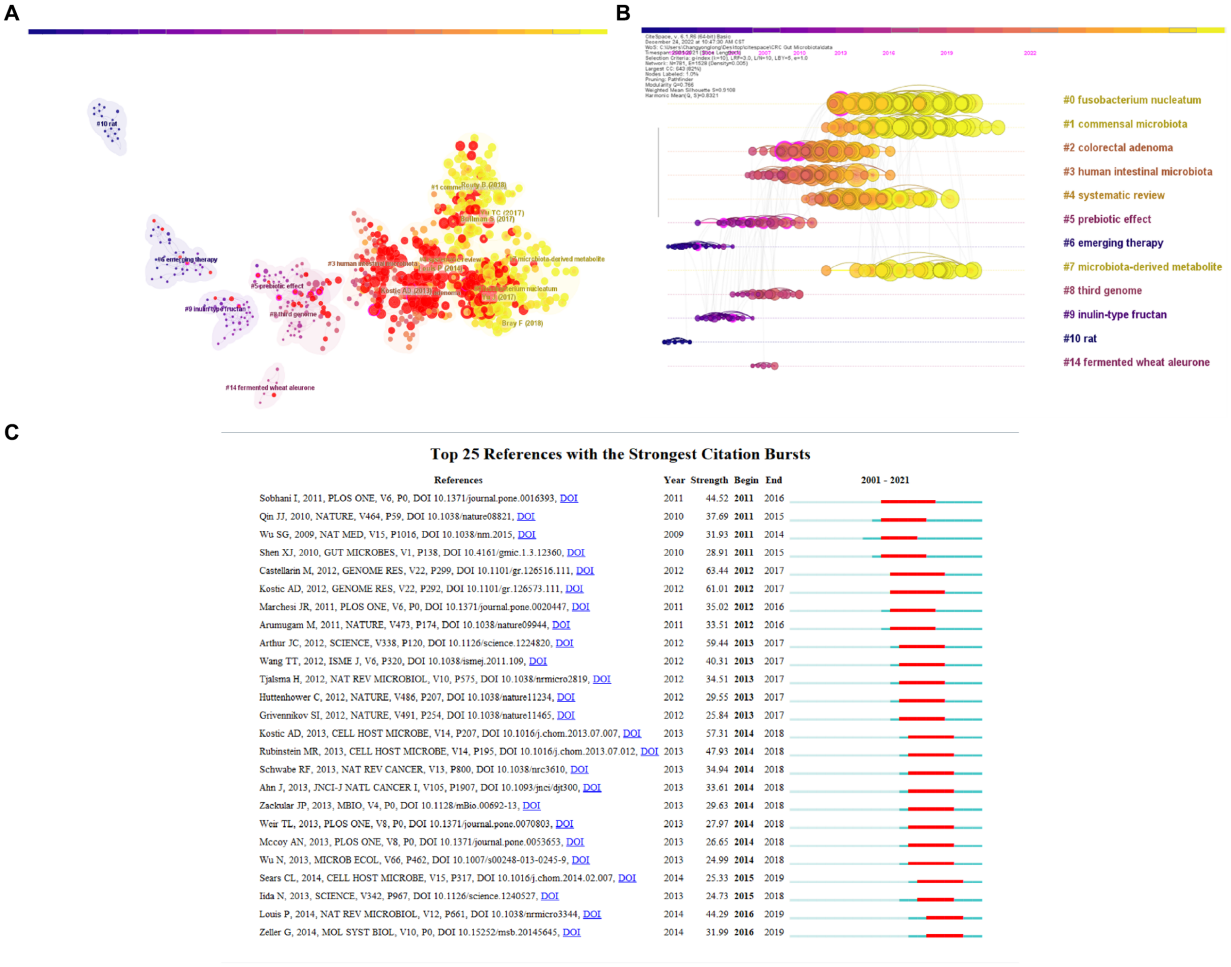
**(A)** CiteSpace uses log-likelihood ratios (LLR) to obtain clustering results and add labels to the co-cited publications. Different patterns represent a cluster. Label # indicates the cluster to which it is assigned, and the smaller the number, the more co-cited studies are in the cluster, **(B)** A timeline visualization of co-cited literature in CiteSpace, and **(C)** CiteSpace Visualization map of the top 25 most cited reference.

The co-citation network clusters in the literature were labeled using the CiteSpace clustering function’s automatic tagging technique. As seen in Figure 5A, the largest cluster was named #0 *fusobacterium nucleatum*, indicating that many studies on CRC and gut microbiota cite literature in this cluster. The cluster numbers are labeled in ascending order from 0, with smaller numbers indicating that more studies are included in the corresponding cluster, highlighting the importance of *fusobacterium nucleatum* in this field of research. In 2012, two studies on the CRC microbiome reported that *Fusobacterium nucleatum*, a common oral anaerobic bacterium, was significantly enriched in cancer tissues (Wang and Fang, 2022). Subsequently, several studies have demonstrated that this bacterium is involved in the initiation, progression, and chemoresistance of human CRC lesions. This indicates that the visualized results are consistent with the development trajectory of this field. Additionally, the brighter color of the clusters, i.e., the color closer to orange, indicates recently published literature in the clusters. Timeline analysis data visualization is a technique that employs clustering and time-based segmentation. Clustering resulted in the organization of the labels, and this visual representation not only displays the topics’ distribution in the field but also reveals trends and connections between research topics over time. The nodes of different colors in the same row in Figure 5B represent different years, and a straight line at the same horizontal position indicates the set of all clustered references, with the cluster labels positioned at the rightmost end of the line. The first three clusters are #0 *fusobacterium nucleatum*, #1 commensal microbiota, and #2 colorectal adenoma.

Figure 5C displays the top 25 most cited references, and it is evident that the first citation burst occurred in 2011. High values in the reference column of scientific mapping studies often signify significant milestones (Du et al., 2022). In this study, the first milestone article was the first large cohort study demonstrating changes in the composition of the microbiota in CRC patients (Sobhani et al., 2011). Furthermore, almost all of the publications on gut flora in CRC focus on articles with reliable citations in the last decade, indicating that the research area may continue to expand in the future.

### Analysis of co-cited keywords

**Figure 6:**
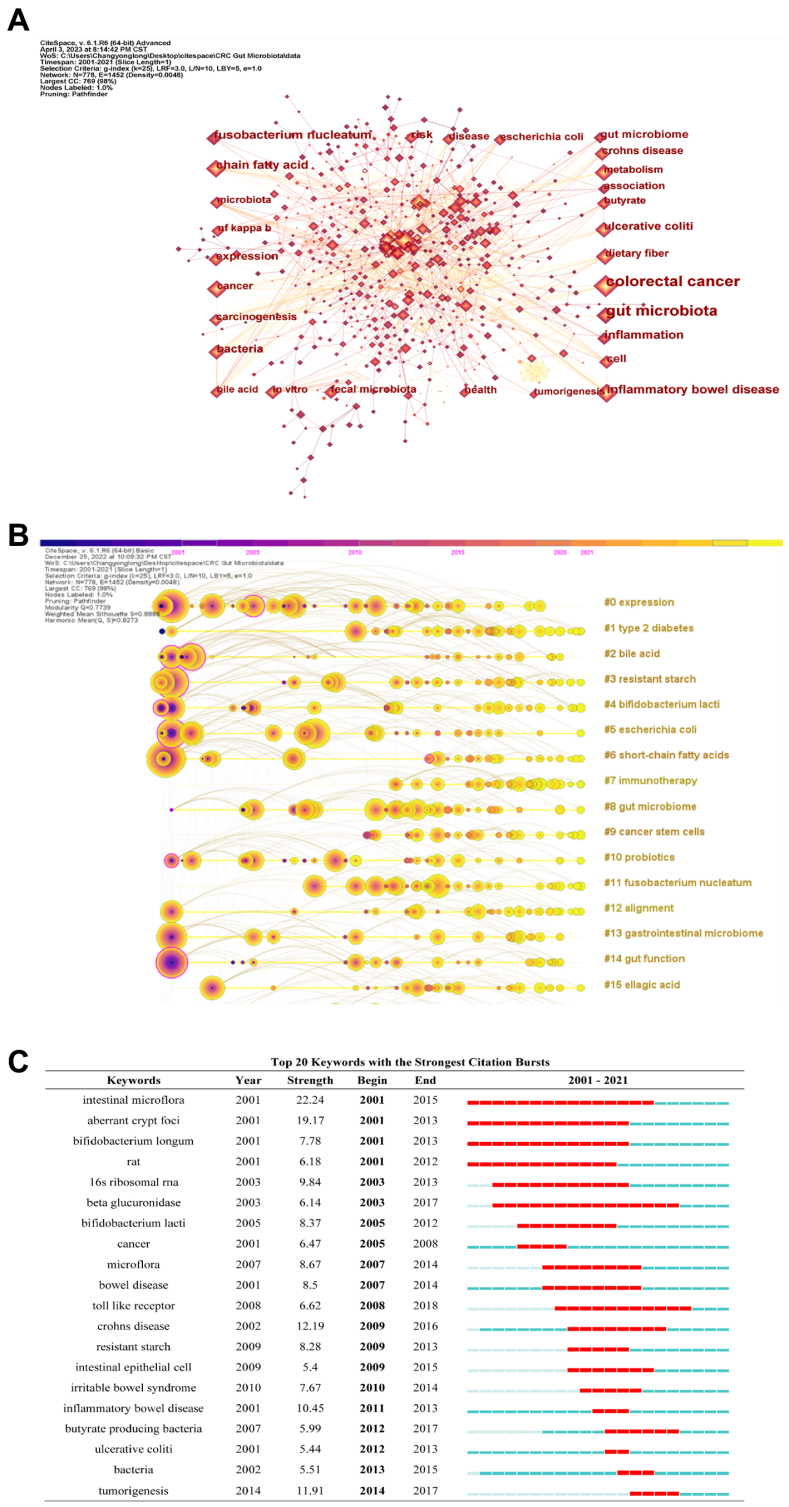
**(A)** CiteSpace visualization of the top 10 most frequently occurred keywords, **(B)** CiteSpace visualization of keyword timeline maps, and **(C)** Top 25 keywords with the strongest citation bursts in this field over the past 20 years. Year indicates the year in which the keyword first appeared, strength indicates the citation burst strength of the keyword, and the end indicates the beginning and ending times of the citation burst of the keyword. Red indicates the period of occurrence of the citation burst.

Keywords reflect the main concepts of an article and studying them can provide a better understanding of the research topic (Wang et al., 2022). Additionally, high-frequency keywords often reflect the research hotspots in the field (Song et al., 2022). In this study, we extracted and analyzed the keywords of 2,707 publications using CiteSpace to obtain keyword co-occurrence network graphs, keyword timeline graphs, and the top 25 keywords with the highest citation intensity. As shown in Table 6 and Figure 6A, among the 778 keywords, the top three keywords used most frequently were inflammatory bowel disease (335 times), *fusobacterium nucleatum* (335 times), and inflammation (305 times), indicating their importance in the field. The keyword timeline graph in Figure 6B shows the top three clusters as #0 expression, #1 type 2 diabetes, and #2 bile acid. Burst detection analysis in Figure 6C reveals that intestinal microflora, aberrant crypt foci, and *bifidobacterium longum* had the highest intensity values, indicating they were highly cited in a specific period of time. These findings demonstrate the importance of these keywords and their association with the research hotspots in the field.

**Table 6:** Top 10 most frequent keywords analyzed using CiteSpace.

Rank	Keywords	Records	Centrality
1	Colorectal cancer	1,762	0.03
2	Gut microbiota	1,436	0.01
3	Inflammatory bowel disease	335	0.07
4	*Fusobacterium nucleatum*	335	0
5	Inflammation	305	0.02
6	Chain fatty acid	304	0.1
7	Bacteria	226	0.11
8	Ulcerative coliti	221	0.03
9	Risk	218	0
10	Gut microbiome	211	0.02

### Country/region collaboration analysis

**Figure 7:**
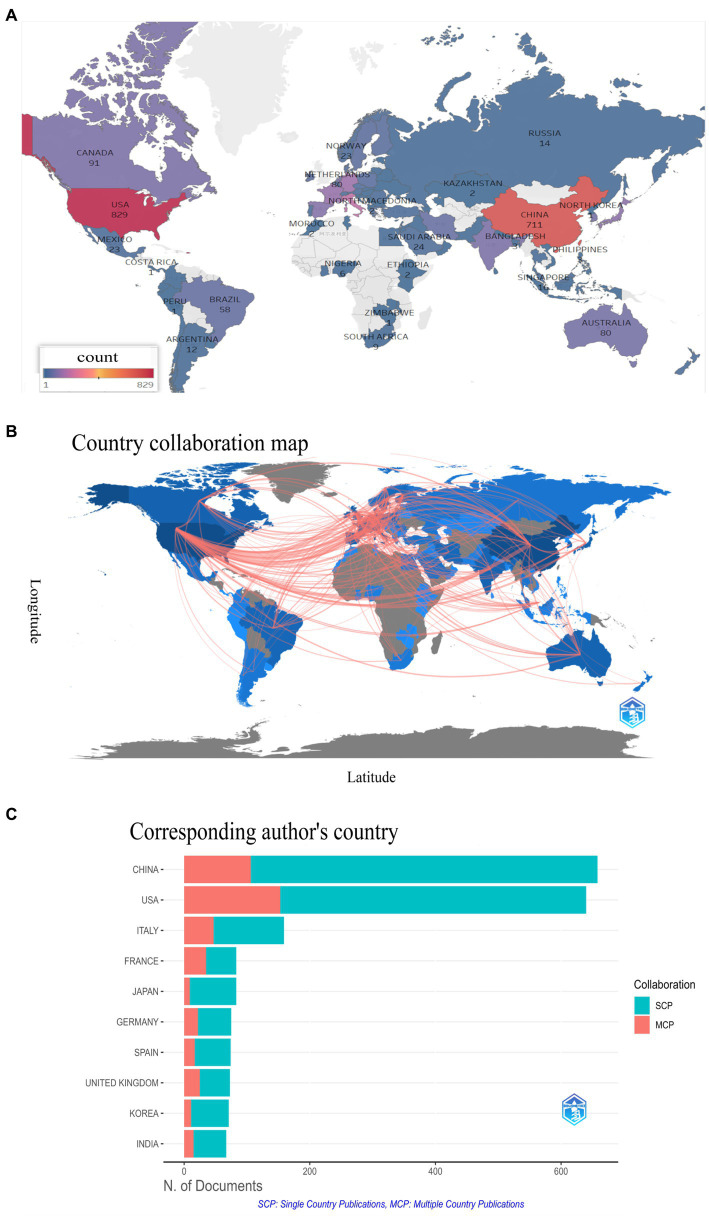
**(A)** Geographical distribution map based on the total number of publications in different countries/regions, **(B)** Global visualization map of publications and collaborative relationships. A red line indicates a collaborative relationship between two countries/regions, and **(C)** Visualization of the countries of the obtained publications’ corresponding authors using biblioshiny.

To identify the countries/regions publishing the articles, we used CiteSpace and imported the data into Tableau Public for visualization. Figure 7A shows that the USA, China, and Italy have the highest number of publications. Figure 7B displays the collaboration network between countries/regions, where a red line indicates collaboration between two countries. We also analyzed the corresponding author’s country/region information using biblioshiny, as shown in Figure 7C. Notably, the USA, despite being ranked second, has the most multiple-country publications (MCP), indicating its collaborative relationships with several countries/regions in this field.

### Top 10 journals with the highest H-index scores

**Figure 8:**
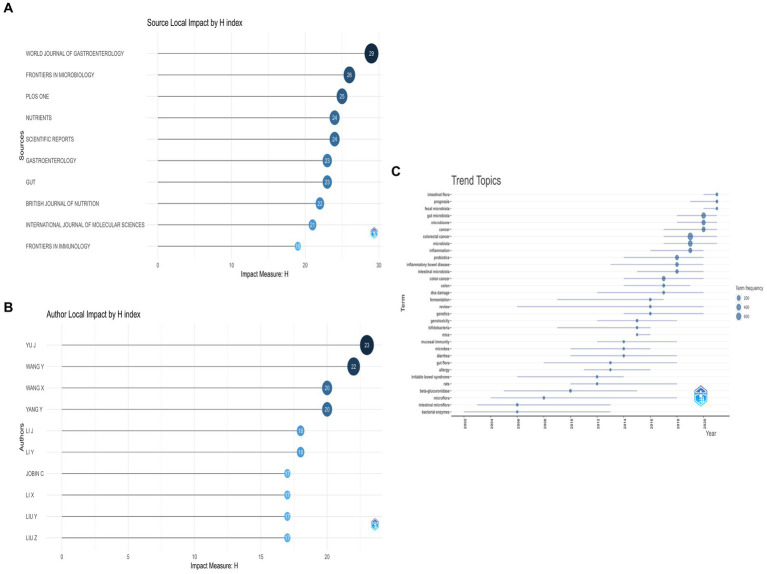
**(A)** Visualization of the top 10 journals with the highest H-index scores using biblioshiny, **(B)** Visualization of the top 10 authors with the highest H-index scores using biblioshiny, and **(C)** Thematic trends on gut flora in CRC.

The H-index is a widely used quantitative metric to evaluate the productivity and impact of scholarly output, proposed by George Hirsch in 2005 (Hirsch, 2005). The H-index reflects the number of papers published by a researcher that have received h or more citations each, and the remaining papers have been cited less than or equal to h times each. When evaluating the scholarly value of a scientific journal, the H-index takes into account both the number of papers published in the journal and the frequency of citations of these papers. In this study, we used the H-index to evaluate the top journals publishing articles on gut flora in CRC research. As shown in Figure 8A and Table 7, the World Journal of Gastroenterology ranked first in terms of the H-index, followed by Frontiers in Microbiology. Interestingly, Gut had the least number of publications among the top 10 journals, yet it was ranked seventh in terms of H-index scores.

**Table 7:** H-index scores of the top 10 journals.

Rank	Journal	h-index	Total citation
1	World journal of gastroenterology	29	2,971
2	Frontiers in microbiology	26	2,221
3	Plos one	25	4,120
4	Nutrients	24	3,076
5	Scientific reports	24	1,471
6	Gastroenterology	23	3,343
7	Gut	23	4,469
8	British journal of nutrition	22	2,947
9	International journal of molecular sciences	21	2,179
10	Frontiers in immunology	19	1,704

### Top 10 authors with the highest H-index scores

The H-index is a quantitative metric used to evaluate the scholarly output of a researcher, based on the number of papers published and the number of times those papers have been cited. Table 8 shows the top ten authors in terms of the H-index and total citation frequency. As shown in Figure 8B, Yu, J. has the highest H-index, followed by Wang, Y. and Wang, X.

**Table 8:** H-index scores of the top 10 authors.

Rank	Author	h-index	Total citation
1	Yu J	23	2,849
2	Wang Y	22	1,720
3	Wang X	20	2,825
4	Yang Y	20	1,573
5	Li J	18	2,527
6	Li Y	18	1,861
7	Jobin C	17	2,605
8	Li X	17	1,488
9	Liu Y	17	1,128
10	Liu Z	17	1,354

### Research trends over the last two decades

We used biblioshiny to visualize the main keywords of publications on enteric flora in CRC over the past 20 years and analyze the research trends. As shown in Figure 8C, each line represents a theme, with the circle indicating the year in which the theme is the most common. A larger circle indicates that the theme appears more frequently. Before 2010, the research hotspots were relatively homogeneous, mainly focusing on beta-glucuronidase and intestinal microflora. However, after 2010, the research hotspots showed rapid growth. The new keywords that appeared over the last 2 years were prognosis and fecal microbiota, indicating that they might be current research hotspots (Xu et al., 2022). The top five themes in terms of total frequency were gut microbiota, inflammation, colorectal cancer, prebiotics/probiotics, and intestinal flora, reflecting the main research directions and hotspots in this field.

## Discussion

In this study, we applied bibliometric techniques and information visualization to comprehensively analyze publications on CRC and intestinal flora from 2001 to 2021. Our analysis adds to the existing literature and provides new insights into this area of research. Based on our findings, the number of scientific publications related to CRC and intestinal flora has increased over the past 20 years, indicating the growing importance of this field. Our study contributes to refining the understanding of this topic and highlights the potential for further research.

Based on the trends observed in the volume of publications, our study analyzed the authorship, institutional affiliations, countries/regions of origin, journals, keywords, co-cited literature, and collaborations at three levels: quantitative analysis, qualitative analysis, and comprehensive analysis. Additionally, we discussed the evolution of research trends in this field. Therefore, several aspects need to be discussed in detail to provide a comprehensive overview of our findings.

### General information

Firstly, the number of publications per year from 2001 to 2015 was relatively stable without much fluctuation. However, after 2015, the annual number of publications showed a rapid increase, which may be attributed to the fact that several research studies have emerged in this field with the advancement in science and technology research. For example, the application of sterile animal and microbial culture techniques, the development of high-throughput sequencing genomics technology, the rise of research in systems biology, and the strong support from the government have further motivated researchers to devote themselves to this field of research (Fong et al., 2020; Zhao et al., 2021; Coker et al., 2022).

Our analysis of publications on CRC and enteric flora over the past 20 years revealed that the USA had the highest number of publications, followed by China and Italy. Among institutions, Shanghai Jiao Tong University, Harvard Medical School, and The Chinese University of Hong Kong had the highest number of publications. Yu, Jun from the Department of Medicine and Therapeutics, State Key Laboratory of Digestive Diseases, LKS Institute of Health Sciences, Shenzhen Research Institute, and The Chinese University of Hong Kong was the author with the most published papers and the highest H-index. PLOS ONE was the journal with the highest number of publications in this field, while the World Journal of Gastroenterology had the highest H-index. Our analysis also showed a disparity between productivity and impact, with some highly productive journals not having a high impact and vice versa. For example, Gastroenterology had a high impact but did not publish a large number of papers, suggesting that it maintains strict quality control over its publications.

### Knowledge base

The concept of co-citation in literature was first introduced by American intelligence scientist Small in 1973. Co-citation occurs when two or more papers are cited together in one or more subsequent papers, indicating a significant relationship between the co-cited papers (Shen et al., 2022). The more frequently a paper is cited, the more significant it is; therefore, highly cited papers are often of particular importance to us. Among the top 10 co-cited papers, Sobhani et al. (2011) have conducted a clinical study demonstrating for the first time that the changes in microbiota composition in CRC patients may affect mucosal immune responses. This study has pioneered new areas of research in large-scale screening of the disease and pathophysiology. Qin et al. (2010) applied Illumina’s metagenomics sequencing technology to sequence total fecal DNA from 124 Europeans and provided a comprehensive view of the human gut microbiome while building an extensive gene catalog, which forms the basis for many studies on gut microbiome function and human phenotype. Wu et al. (2009) from the Department of Medicine, Johns Hopkins University School of Medicine, Baltimore, MD, USA, investigated the immune mechanism of colorectal carcinogenesis caused by a common human colon bacterium, namely, ETBF. They demonstrated that ETBF-induced colon cancer is dependent on Stat3 and TH17, thereby identifying a unique role of adaptive immunity in the pathogenesis of colon cancer. Shen et al. (2010) applied molecular methods to conduct a cross-sectional study of adherent bacteria in the normal colonic mucosa of 21 colorectal adenomas and 23 non-adenoma subjects. The results from that study indicated an altered bacterial community composition associated with colorectal adenoma when compared to that in the control. This indicated that adherent bacteria may play an important role in the development of colorectal adenomas and CRC, thus providing a therapeutic strategy for microbiota control to prevent colorectal adenomas and cancer and to identify high-risk individuals. *Fusobacterium nucleatum*, a strict anaerobic gram-negative bacterium, is widely distributed in the oral cavity, upper respiratory tract, and vagina. It is often associated with periodontal disease, appendicitis, IBD, respiratory infections, rheumatoid arthritis, cardiovascular disease, chorioamnionitis, preterm birth, stillbirth, Alzheimer’s disease, etc. A study by Castellarin et al. (2011) from the Canadian Cancer Institute in 2011 confirmed that *Fusobacterium nucleatum* is also present in the intestine, with high abundance in the colon. There is a close relationship between the abundance of this bacterium and CRC. This revealed that *Fusobacterium nucleatum*-related biomarkers are the indicators of CRC or the risk of CRC. In 2011, another research team from the Broad Institute of MIT and Harvard University also showed the presence of *Fusobacterium nucleatum* in CEC tissues, with results similar to those of Castellarin et al. Kostic et al. (2011) performed DNA analysis of *Fusobacterium nucleatum* and demonstrated that the relative abundance of *Fusobacterium nucleatum* was much higher in tumor specimens than in normal tissues. Further, they evaluated the relationship between *Fusobacterium nucleatum* and the survival and maintenance of tumor cells and observed that *Fusobacterium nucleatum* may be significantly associated with the pathogenesis of certain subtypes of CRC. In addition, they observed that *Fusobacterium nucleatum* was the most dominant strain in cancer tissues; however, more than one dominant strain was present in several tumors. They concluded that the presence of multiple strains in some tumor patients indicates the involvement of all these microbes in tumor formation, with the most dominant mechanism likely to be inflammation-mediated. Marchesi et al. (2011) through deep rRNA sequencing techniques compared the microbiota of six cases of colon cancer tissue and adjacent non-malignant mucosa thus providing the first high-resolution image of the human CRC microbiome and showing that CRC is associated with rather significant changes in the adherent gut microbiota. Arumugam et al. (2011) identified three characteristic clusters by metagenomic analysis of 22 fecal samples from 4 countries and called them enterotypes. Their results concluded that enterotypes are determined by strain composition, but important functions are not necessarily determined by the species in large amounts, and that enterotypes are independent of host BMI, age, and country. Also, the study identified some gene modules associated with age and BMI that could be used as microbial markers for diagnostic purposes in the future. This article introduced the concept of “enterotype” for the first time, and although many scientists have since questioned it, the concept of “enterotype” still has its influence today. Arthur et al. (2012) applied high-throughput sequencing technology in a mouse model of colorectalitis and found that inflammation can create an environment that supports carcinogenesis by affecting the host and microbiota. Thus, this study emphasized the complex effects of inflammation on the microbial composition/activity and the ability of the host to protect itself from adverse microbiota. Butyrate, if present in adequate amounts, can be involved in the protection against colitis and CRC by reducing oxidative DNA damage, inducing apoptosis in DNA-damaged cells, inhibiting tumor cell growth, and reducing co-carcinogenic enzyme activity. The depletion of butyrate-producing bacteria in the gut microbiota leads to a structural imbalance in the gut microbiota, as evidenced by a decrease in butyrate production and an increase in the number of opportunistic pathogens. Wang et al. (2011) from the State Key Laboratory of Microbial Metabolism, School of Life Sciences and Biotechnology, Shanghai Jiao Tong University, compared the gut microbiota composition of CRC patients and healthy volunteers in a cross-sectional cohort study and observed structural imbalances in the gut microbiota of CRC patients. The study further revealed that structural imbalances in the gut microbiota may provide new insights for an in-depth analysis of host–microbe interactions and for determining their role in cancer development.

Overall, these ten high-frequency co-cited articles demonstrated the exploration and development of intestinal flora in CRC, including further disease typing, determination of potential diagnostic markers, application of high-throughput sequencing technologies, the association of flora and inflammation as well as flora and immunity, and the relationship between changes in the structure of flora and the development of the disease. The analysis of these co-cited articles can provide us with a large amount of useful information to better understand the evolution of the research on the role of intestinal flora in the prevention, diagnosis, and treatment of CRC.

### Emerging topics

Keywords highlight the research themes and core content. An analysis of keyword co-occurrences can provide insight into the spread and growth of research topics in a particular field. Here, we used CiteSpace to construct the keyword co-occurrence network graph, construct the keyword clustering timeline graph, and determine the top 25 keywords with the strongest citation bursts. In addition, we applied the bibliometric package biblioshiny function in R for visualizing the keywords of publications over the last 20 years to analyze the hotspots and development trends of research on gut flora in CRC.

High-frequency keywords in the keyword co-occurrence network diagram and a timeline view of keyword clustering (Figures 8A,B) mainly include inflammatory bowel disease (Guarner and Malagelada, 2003) *fusobacterium nucleatum* (Mármol et al., 2017), inflammation (Holmes et al., 2011; Feng et al., 2015), long-chain fatty acids (Roberfroid et al., 2010; Louis et al., 2014) ulcerative colitis (Fukata et al., 2007; Marchesi et al., 2015), bile acid (Feng et al., 2015), and resistant starch (Conlon and Bird, 2014). This indicated that intestinal flora and digestive system-related diseases especially IBD are the hotspots in research on intestinal flora in CRC (Schwabe and Jobin, 2013). In addition several metabolites play key roles in the development of intestinal flora in CRC (Kaczmarczyk et al., 2012; Zeller et al., 2014; Diether and Willing, 2019). The analysis of top keywords using burst detection (Figure 8B) indicated that in the last two decades aberrant crypt focibifidobacterium (Ridlon et al., 2016; Routy et al., 2018), beta-glucuronidase lactic acid bacteria 16 s ribosomal RNA microflora toll-like receptor (Koropatkin et al., 2012), Crohn’s disease and butyrate-producing bacteria have attracted much attention from the researchers in this field (Farrell and Peppercorn, 2002). The research focus of intestinal flora in CRC has shifted from a single bacterium to flora omics from flora monograph to combined analysis of flora metabolism and other multi-omics from traditional techniques to 16S RNA sequencing and metagenomics and from basic research to clinical applications. As shown in Figure 8B. methylator phenotype was one of the widely studied topics (Trinchieri, 2012). In recent years epigenetic alterations of DNA methylation, i.e., CIMP (Zhao et al., 2021), have been widely studied. Recent research has suggested that the gut microbiome may be associated with cancer development spread and metastasis through epigenetic mechanisms such as DNA methylation (Brennan and Garrett, 2016) histone modifications and non-coding RNA. This provides an epigenetic view of the relationship between the gut microbiome and CRC. Multiple studies have indicated comprehensive and convincing evidence linking the gut microbiota to CRC at the epigenetic level including the oncogenic mechanisms of cancer-associated microbiota and the use of the gut microbiota as a potential biomarker for CRC (Hajishengallis et al., 2012; Johansson and Hansson, 2016). In a cohort study of 1,000 CRC patients, Sobhani et al. (2019) observed that Wif1 PENK and NPY gene promoters were hypermethylated in CRC patients and the cumulative methylation index (CMI) of these genes was significantly higher than in that in the controls. Thus it was concluded that dysbiosis associated with CRC patients induces host gene methylation and the corresponding CMI and associated bacteria are potential biomarkers for CRC

In conclusion, the clustering analysis of keyword co-occurrence network and keyword timeline graphs indicated that the research on gut flora in CRC over the past two decades has focused on the following five areas: the role and mechanism of gut flora in CRC, the impact of structural imbalance of flora on CRC, metagenomics, the application of 16S RNA sequencing technology, the combined application of gut flora and metabolomics in CRC, and finally the application of gut flora as biomarkers in CRC (Nenci et al., 2007; Rubinstein et al., 2013; Bonder et al., 2016).

## Limitations

Compared to traditional approaches, the visual analysis techniques of CiteSpace, VOSviewer, and R provide a more comprehensive understanding of the evolving research focus and trends regarding the relationship between gut flora and CRC. However, this study has limitations. The publications were only searched for in the core dataset of the Web of Science database, and only English literature was included, which may have resulted in some original literature being missed. Therefore, the conclusions may not be comprehensive. In the future, we plan to continue our research in this field by collecting more research data and enriching our findings to provide valuable information and assistance to researchers.

## Conclusion

There is no doubt that gut flora has significant research potential and therapeutic prospects in CRC. This study provides a comprehensive metrological and statistical analysis of research on gut flora in CRC over the past 20 years. In this study, we used visualization tools such as CiteSpace and VOSviewer to analyze 2,707 publications during 2001–2021 and observed an increase in the number of publications in international core journals, and the USA and China are the most important countries of origin of publications. Besides, researchers from several countries and institutions are conducting research and building collaborative relationships in this field. The USA remains the major source of highly cited literature in this field. Future research in this field will focus on utilizing probiotics, utilizing gut flora as biomarkers for CRC, and developing gut flora as therapeutic targets.

## Data availability statement

The original contributions presented in the study are included in the article/Supplementary material, further inquiries can be directed to the corresponding author.

## Author contributions

SZ designed this study and revised the manuscript. YC conducted the data collection, bibliometric and statistical analysis, figures visualization and manuscript writing. All authors contributed to the article and approved the submitted version.

## Funding

This work was financially supported by the Research projects of traditional Chinese medicine of Hunan Province (No. A2023043), and Hunan Provincial Health and Family Planning Commission Research Project (No. 20200424).

## Conflict of interest

The authors declare that the research was conducted in the absence of any commercial or financial relationships that could be construed as a potential conflict of interest.

## Publisher’s note

All claims expressed in this article are solely those of the authors and do not necessarily represent those of their affiliated organizations, or those of the publisher, the editors and the reviewers. Any product that may be evaluated in this article, or claim that may be made by its manufacturer, is not guaranteed or endorsed by the publisher.

## Footnotes

^1^ http://127.0.0.1:6491/

## Abbreviations

CRC: Colorectal cancer
IBD: Inflammatory bowel disease
Wos: Web of Science
WoSCC: Web of Science Core Collection
ETBF: Enterotoxigenic *Bacteroides fragilis*
IF: Impact factor
