# Supplementary material for: Global research trends and focus on the link between colorectal cancer and gut flora: a bibliometric analysis from 2001 to 2021
Source: Front Microbiol. 2023 May 5;14:1182006. doi: 10.3389/fmicb.2023.1182006 (PMC10196369; doi:10.3389/fmicb.2023.1182006)
Supplement: Supplementary file 1 [file Table_1.DOCX]

**Supplementary Table 1. H-index scores of the top 10 authors**

| Rank | Author | h_index | Total citation |
| --- | --- | --- | --- |
| 1 | YU J | 23 | 2849 |
| 2 | WANG Y | 22 | 1720 |
| 3 | WANG X | 20 | 2825 |
| 4 | YANG Y | 20 | 1573 |
| 5 | LI J | 18 | 2527 |
| 6 | LI Y | 18 | 1861 |
| 7 | JOBIN C | 17 | 2605 |
| 8 | LI X | 17 | 1488 |
| 9 | LIU Y | 17 | 1128 |
| 10 | LIU Z | 17 | 1354 |
